# 1370. Prevalence and predictors of post-covid-19 (long covid) in India

**DOI:** 10.1093/ofid/ofad500.1207

**Published:** 2023-11-27

**Authors:** Vignesh Kumar, Jency Maria Koshy, S Divyashree, Suneetha Narreddy, Priscilla Rupali, Sowmya Sathyendra

**Affiliations:** Christian medical college, Vellore, Tamil Nadu, India; Believers church medical college hospital, Thiruvalla, Kerala, India; New Bombay Hospital, mumbai, Maharashtra, India; Apollo Health City, Hyderabad, Telangana, India; Christian Medical College, Vellore, Tamil Nadu, India; Christian Medical College, Vellore, Tamil Nadu, India

## Abstract

**Background:**

Long COVID is a condition that occurs in individuals with a history of probable or confirmed SARS-CoV-2 infection, usually 3 months from the onset of COVID-19, with symptoms that last for at least 2 months and cannot be explained by an alternative diagnosis. This Indian study followed up patients after an episode of acute COVID-19 for 1 year after hospital discharge from different parts of India.

**Methods:**

This was a multi-centric bi-directional study among patients with confirmed COVID-19 which assessed patients at different time points, i.e. 6 weeks, 3-6, 6-9 and 9-12 months. Patients aged ≥18 years with laboratory confirmed COVID-19, were recruited and followed up telephonically using standardized surveys for quality of life. Data on demographics, pre-existing co-morbidities, risk factors, signs and symptoms and hospital parameters during acute COVID-19 infection were noted. An effort was made to include people with different severity of COVID-19. Univariate and multivariate analysis were performed to determine predictors of long covid.

**Results:**

A total of 315 patients were enrolled (Table 1). Of these, 59.4 were males and 40.6 were females. The mean age was 51.98 ± 14.619 years. At 6 weeks and 12 months, 16.5% and 24% reported continuing symptoms. While shortness of breath was common at each time point, persistent muscle pain and weakness waxed and waned (Figure 1). At 1 year of follow-up, 24% still reported at least 1 troubling symptom and 26% reported > 1 symptom that affected their quality of life i.e., nearly 8% of patients experienced problems in doing their usual activities, 11.7 % were slightly anxious or depressed and few 3.5% experienced slight pain or discomfort. In the multivariable analyses, the severity of disease at baseline (OR 2.330, 95% CI 1.080-5.027) and higher education (OR 0.437, 95% CI 0.241-1.793) seemed to be associated with long COVID.

Demographic and clinical characteristics according to the Severity of COVID

**Figure:**
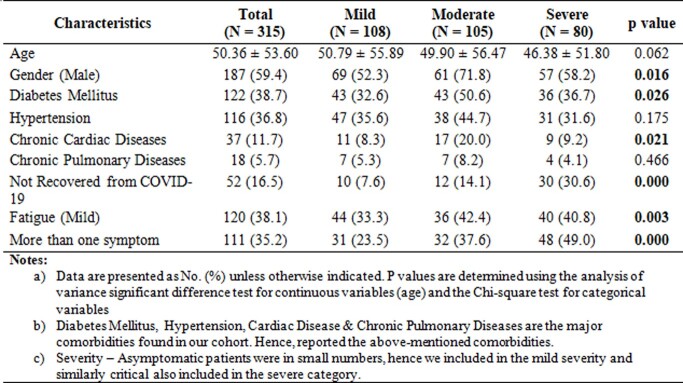

Persisting symptoms of Long COVID

**Figure:**
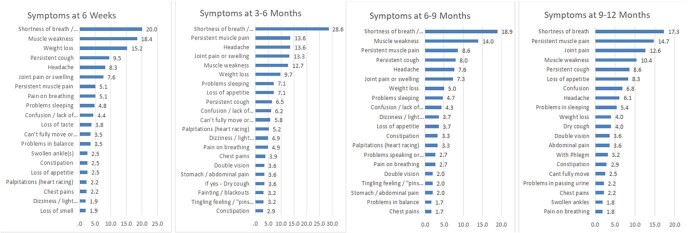

**Conclusion:**

We found that long covid symptoms may be persistent, following an initial recovery from an acute COVID-19 and may also fluctuate or relapse over time. Since symptoms persist in at least 25% of patients for at least 1 year after discharge, we urgently need therapeutic interventions which can improve the quality of life in patients after an acute episode of COVID-19 infection.

**Disclosures:**

**All Authors**: No reported disclosures

